# Advancing Surgical Precision in Z-Plasty and Melanoma Excision Through Quality Improvement Initiatives in Rural Settings

**DOI:** 10.7759/cureus.73691

**Published:** 2024-11-14

**Authors:** Dilothi Selvarajah, Amanpreet K Jolly, Tanreet Pabla, Jaheim Thomas, Adam Dubrowski

**Affiliations:** 1 Human Health Science, Ontario Tech University, Oshawa, CAN; 2 Public Health, Ontario Tech University, Oshawa, CAN; 3 maxSIMhealth Group, Ontario Tech University, Oshawa, CAN

**Keywords:** 3d printing, additive manufacturing, cancer, medical education, melanoma, silicone, simulation, z-plasty

## Abstract

Rural healthcare provider shortages have a severe impact on Canadians who seek care in rural and remote (R&R) settings, often arriving with poor health or medical emergencies. Healthcare providers working in such settings often face significant challenges in accessing skills development and maintenance courses to meet the unique medical demands of rural communities. As a result, it is vital to provide R&R healthcare providers with the appropriate simulation-based skills training. This approach led to the development of a Z-plasty and melanoma simulator tool, which was presented at a workshop during the Society of Rural Physicians of Canada (SRPC) conference in Niagara Falls, Ontario, from April 20 to 22, 2023. The workshop aimed to familiarize participants with the procedures and instruments required for Z-plasty and melanoma excisions in R&R practice. This paper describes the development of the simulators used in the foundational skills workshop, attended by medical students, residents, and physicians. It also analyzes the workshop’s findings to guide future enhancements. The Z-plasty and melanoma simulators were created using additive manufacturing techniques, including three-dimensional printing and silicone. Participants in the SRPC Rural and Remote Medicine Course evaluated the functionality and realism of the simulators and provided feedback for improvements, using the Michigan Standard Simulation Experience Scale. Quantitative data indicated that the Z-plasty simulator achieved an overall score of 4.03 on a 5-point Likert scale, while the melanoma simulator scored 4.15. Participants’ feedback was categorized into three main areas: self-efficacy, realism, and educational value. Qualitative analysis of the data revealed three themes for the Z-plasty simulator: physical resilience, materials science, and skills development. Similarly, the melanoma simulator yielded two main themes: physical reliance and materials science. Overall, the simulators demonstrated effective hands-on practice, representing a sustainable method for developing skills-based competencies in Z-plasty and melanoma excisions for R&R settings. Feedback from workshop participants will inform ongoing improvements to the simulators and their integration into future training events.

## Introduction

Rural healthcare provider shortages have a negative impact on Canadians who seek care in rural and remote (R&R) settings, often arriving with poor health or medical emergencies. Additionally, factors such as distance from urban centers and associated costs make it difficult for healthcare providers who remain in R&R areas to access essential skills development and maintenance courses required to meet the diverse medical needs of rural communities. As a result, it is critical to offer the necessary simulation-based skills training to R&R healthcare providers. Conferences, courses, workshops, and other forms of continuing medical education serve as vital platforms for healthcare providers to stay updated on the latest knowledge, skills, and best practices essential for effective clinical practice [1]. Through simulation-based education (SBE), healthcare providers can hone and master clinical skills, refining their proficiency through repeated practice and hands-on experience [1]. 

Simulation serves as a valuable learning tool that can be applied across various disciplines and by different types of learners. It involves creating immersive and interactive experiences that replicate significant elements of the real world. Through SBE, healthcare professionals can enhance their knowledge, skills, and attitudes while ensuring patient safety by minimizing avoidable risks [2].

However, due to financial and availability constraints, implementing and maintaining SBE in R&R areas can be challenging. Despite these obstacles, leveraging SBE can serve as a valuable strategy to provide hands-on learning experiences. Nevertheless, healthcare providers in R&R areas face significant challenges, such as the costs associated with simulators and the limited access to guidance and feedback [1]. For healthcare practitioners who struggle to access skills development and maintenance courses, SBE becomes a crucial alternative. Using animal models, such as a pig’s foot for practicing wound closure techniques, is one possible approach to SBE [3]. However, this method is far from optimal and raises ethical concerns. A more effective solution is to utilize additive manufacturing (AM) techniques, particularly three-dimensional (3D) printing, to produce cost-efficient and customizable simulators [4]. This approach allows learners to practice and refine their procedural skills at any time, while also enabling the customization of simulators to suit specific contexts. By leveraging AM, expenses can be reduced, and a more ethical and sustainable mode of training can be adopted, moving away from the use of animal products [1]. 

An example of this technique was employed to create a Z-plasty suture simulator and melanoma excision simulator, which were featured in a workshop conducted at the 30th Annual Rural and Remote Medicine Course hosted by the Society of Rural Physicians of Canada (SRPC) in Niagara Falls, Canada, from April 20 to 22, 2023. The SRPC serves as the national voice of rural physicians in Canada, with the primary objective of promoting rural generalist medical care through teaching, collaboration, advocacy, and research. The work of the SRPC encompasses a wide range of initiatives, including the development and promotion of healthcare delivery mechanisms, providing support for rural physicians and communities in need, advancing continuing medical education for rural practitioners, organizing and facilitating research on rural healthcare issues, and fostering collaboration between rural physicians and other groups interested in rural healthcare [5].

Z-plasty is a technique employed in plastic and reconstructive surgery to modify scars through a transposition flap [6]. While this procedure is commonly performed by plastic surgeons, approximately 25 million people lack direct access to these specialists. Many of these individuals live in rural areas, where the absence of specialized care can result in less-than-ideal treatment options. Physicians are more inclined to work in rural areas if they have prior personal experiences in such settings, yet residency programs offer limited exposure to rural plastic surgery. Targeted training in this procedure, specifically for rural practice, could significantly improve patient care in these underserved areas [7]. Knowledge of histological and vascular anatomy, as well as the biomechanical properties of the skin, is imperative in flap surgery. A notable advantage of Z-plasty is that it may eliminate the need for skin removal if the outer layer of the skin covering the scar looks visually pleasing and meets the desired standards for reconstruction. This is achieved by making incisions along the Langer lines, which follow the natural orientation of the dermal collagen fibers. This method has been successfully applied to various body parts, including but not limited to the fingers, nose, chest, palate, face, eye, ear, and numerous others, as well as for releasing scar contracture after burns [6].

Melanoma is a tumor that develops when melanocytes undergo malignant transformation. Proficiency in melanoma excision is essential for effective treatment. A study evaluating medical students, junior doctors, surgical registrars, and plastic surgery consultants assessed their ability to accurately outline melanoma lesions. While surgical registrars performed the best, their accuracy was only around 46%, with other participants scoring even lower. These findings highlight the need for more hands-on training in melanoma excision to improve accuracy in this critical procedure [8]. Melanocytes originate from the neural crest, which is why melanomas can develop in any area where neural crest cells migrate, such as the gastrointestinal tract and the brain, although they do typically occur on the skin [9]. The incidence of melanoma is rising worldwide, with the majority of cases detected at an early stage. However, unlike many other cancers, the death rate for melanoma has remained relatively stable, due to decreasing mortality among younger people and increasing mortality among elderly people. Surgery is still the backbone of treatment for primary melanomas, and it is curative in many cases. Appropriate surgical management is crucial for diagnosing, staging, and effectively treating invasive primary cutaneous melanoma [10]. The goals of surgery are to obtain histologic confirmation of the diagnosis, accurately perform micro-staging, and ensure proper excision of the margin around the original site to minimize the risk of local recurrence. [10]. Since surgery is the primary method of melanoma treatment, AM offers the potential to produce melanoma simulators that can be used to train healthcare providers in the surgical skills necessary for effective cancer treatment. 

The goal of this technical paper is to describe the development of the Z-plasty and melanoma simulators and provide initial user-driven evaluations from a skills workshop hosted by the SRPC, which aimed to provide hands-on training to R&R healthcare providers. Simulation-based training offers healthcare professionals a valuable chance to apply theoretical knowledge and develop technical skills in complex procedures, all while minimizing risks to patient safety [11]. This approach is particularly beneficial for medical students and residents, as it provides one of the most effective ways to gain hands-on experience in a controlled setting.

## Materials and methods

The research ethics for this study was exempted by the Ontario Tech University Research Ethics Board under the reference REB-17296. 

The contents of this paper detail the progression of the development, feasibility assessment, and pilot testing of a modified version of the Medical Research Council (MRC) framework [12]. In general, the MRC framework provides a structured approach for designing and evaluating complex interventions, including tools for medical education such as simulators. During the development phase, the MRC framework is applied to define the specific components that a simulator needs to fulfill in alignment with learning objectives. In the feasibility and pilot stages, the primary objectives are to evaluate the simulator's feasibility, acceptability, and initial effectiveness. Within this stage, a small cohort of learners engages with the simulator to identify potential technical issues, user experience drawbacks, and potential areas for enhancement. Insights from both learners and instructors are collected to iteratively refine the simulator’s design [12]. 

The workshop employed a mixed-methods research approach, which integrates both qualitative and quantitative data collection to address research questions or hypotheses [13]. This approach utilizes rigorous methods for data collection, analysis, and interpretation of both types of data. It is chosen for its ability to leverage the strengths of both qualitative and quantitative research while addressing their respective limitations [13]. Our survey applied this method by incorporating quantitative questions using the Likert scale alongside qualitative inquiries. 

Simulator development 

Z-Plasty

**Figure 1:**
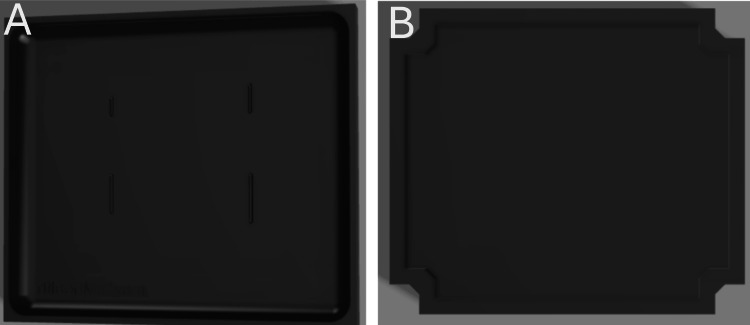
3D-rendered design of the Z-plasty simulator mold in Fusion 360™. (A) Mold for the skin layer with scars. (B) Mold for the muscle layer.

**Figure 2:**
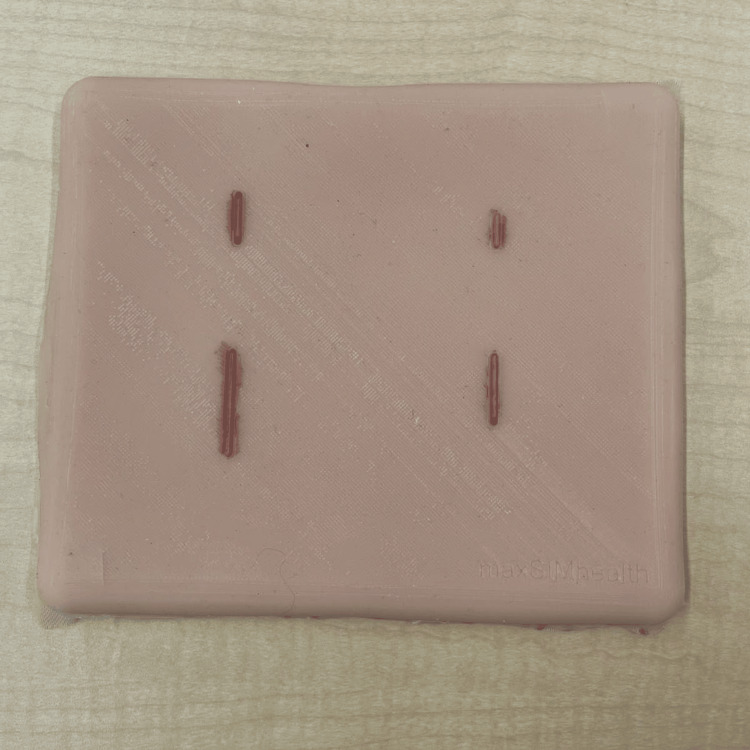
The Z-plasty simulator.

The design of the Z-plasty simulator was developed in collaboration with the instructors who conducted the workshop at the SRPC. They indicated the need for a simulator with varying sizes and depths of Z-plasty incisions. Based on this, the Z-plasty simulator was designed using Fusion 360™ (Autodesk Inc., San Rafael, CA, USA), to be produced in two parts: one representing the muscle layer and the other representing the skin layer with a small scar where the incision would be made, as shown in Figure 1. This design approach ensured the skin layer could be manipulated to accurately simulate the procedure. The design was then 3D-sliced using UltiMaker Cura 3D printing software (UltiMaker B.V., Utrecht, The Netherlands) and 3D-printed with EcoTough™ PLA filament (FILAMENTS.CA, Kitchener, ON, Canada) on an UltiMaker S5 3D printer. Ecoflex™ 00-20 FAST silicone (Smooth-On, Macungie, PA, USA) was used for both the skin and muscle layers of the Z-plasty simulator, with power mesh (80% nylon/20% spandex; Green Brook, NJ, USA) inserted between the skin layers to increase durability in the areas where incisions would be made. The skin layer was colored with various skin tones using Silc Pig™ (Smooth-On, Macungie, PA, USA) with red silicone coloring applied to the scars, and the muscle layer was colored with red Silc Pig™. Each layer was made separately and then combined by sealing the edges with Ecoflex™ 00-20 FAST silicone. The resulting simulator is shown in Figure 2. In total, 20 Z-plasty simulators were produced, costing $240 for materials, or $12 per simulator. 

Melanoma 

**Figure 3:**
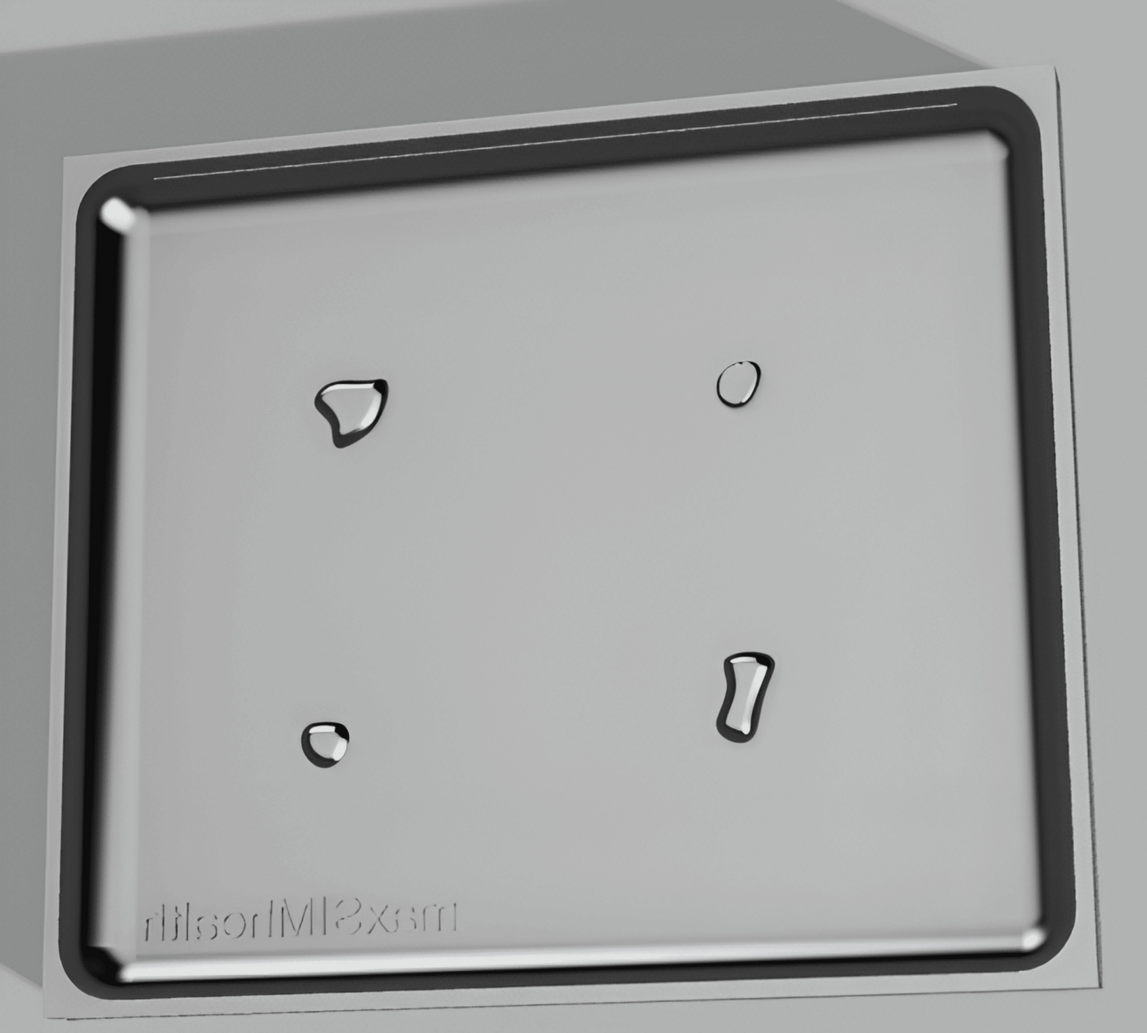
3D-rendered design of the melanoma simulator mold in Fusion 360™.

**Figure 4:**
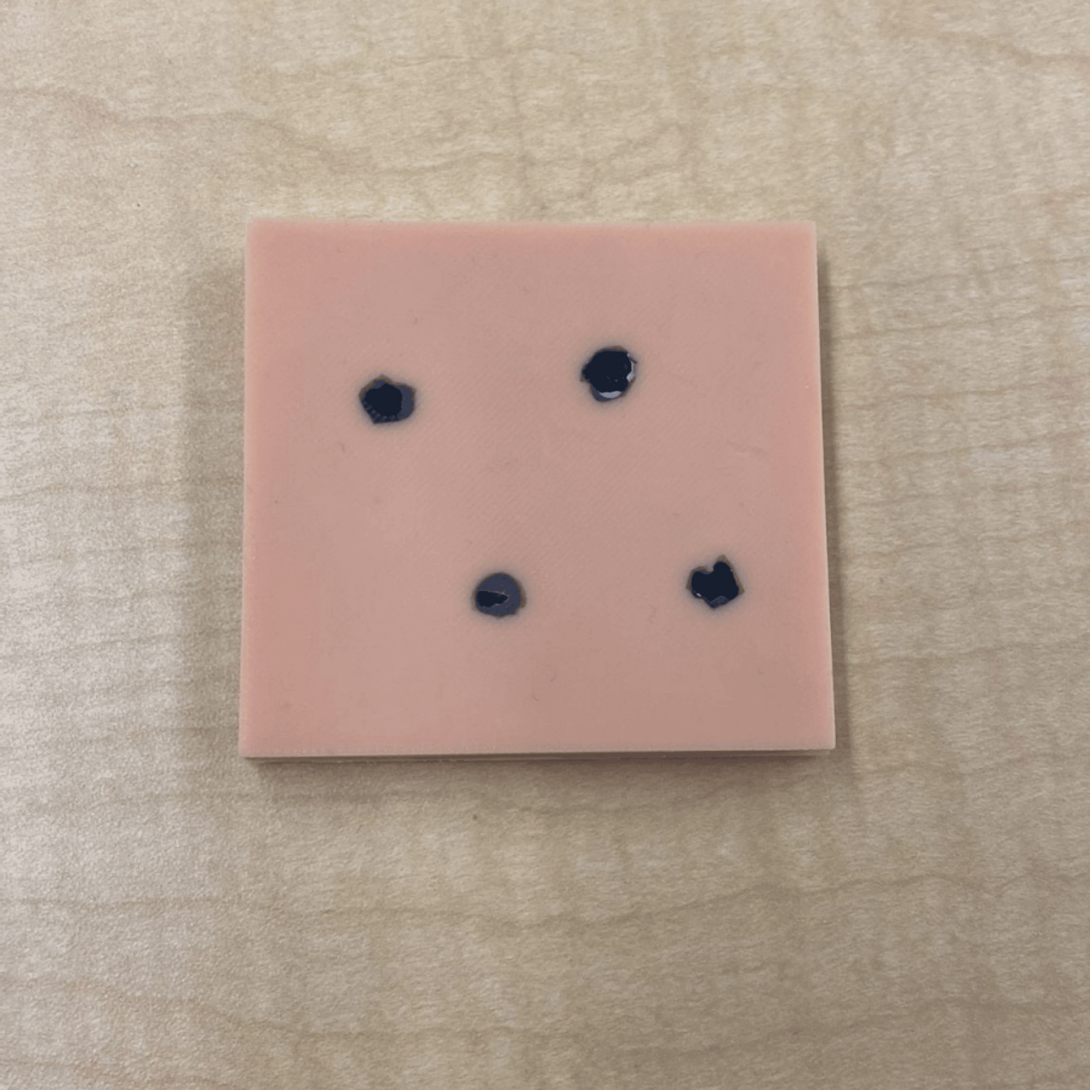
The melanoma simulator.

The design of the melanoma simulator was developed in collaboration with the instructors who conducted the workshop at the SRPC. They indicated the need for melanomas with varying shapes, sizes, and depths. Based on this, the melanoma simulator was designed using Fusion 360™ to represent a rectangular skin pad with indents shaped like various melanomas, each with different shapes and depths, as shown in Figure 3. The design was 3D-sliced in UltiMaker Cura 3D printing software and 3D-printed with EcoTough™ PLA filament on an Ultimaker S5 3D printer. Ecoflex™ 00-20 FAST silicone was used as the base of the melanoma simulator, with power mesh (80% nylon/20% spandex) inserted between the layers to increase the durability of the simulator. The base was colored with various skin tones using Silc-Pig™. To create the melanomas, Dragon Skin™ 10 NV silicone (Smooth-On, Macungie, PA, USA), colored in black/brown Silc-Pig™, was poured into the indents within the base of the melanoma pad, as shown in Figure 3. Twenty melanoma simulators were produced, costing $240.00 CAD for materials, or $12.00 CAD per simulator. Figure 4 shows the final product. 

Participants 

In the advanced wound assessment and suturing workshop at SRPC, 14 participants (n = 14), including medical students, residents, and doctors, attended the Z-plasty session, while 12 participants (n = 12) joined the melanoma excision session. The workshop included individuals from various stages of training and from different specialties, all of whom registered specifically for these sessions. Members of the research team (DS and AJ) were present to explain the study, assist with consent form completion, collect the survey data, and inform the participants that their simulator evaluation results might be used in future publications.

Procedures

The workshop was held in a conference room. The simulator and the tools necessary to perform the specific techniques were placed on a table in front of each participant. The instructors began by reviewing the fundamental principles of suturing. They then guided the participants through the process of performing the techniques on the simulators. The participants then had the opportunity to practice the techniques on the simulator. They were also given the opportunity to ask the instructors for any feedback on their skills throughout the session. Following the workshop, the participants were asked to complete a survey regarding their experience using the simulator. 

Data collection methods 

The evaluation of the two simulators was conducted using a single cohort, post-test design. This approach involved assessing participants' experiences and outcomes after they had completed the simulation. To gather a comprehensive understanding of the simulator's effectiveness and realism, both quantitative and qualitative data were collected. Quantitative data were obtained through structured surveys, which provided measurable insights into participants' knowledge, skills, and satisfaction levels. Additionally, qualitative data were gathered through open-ended questions embedded in the survey and brief interviews, allowing for an in-depth exploration of participants' perspectives, experiences, and suggestions for improvement. This mixed-methods approach ensured a robust evaluation, capturing a wide range of data to inform the effectiveness and potential areas for enhancement of the simulator [13].

After the workshop, the participants were asked to complete a paper-based survey derived from the Michigan Standard Simulation Experience Scale, aimed at gathering insights on three key aspects of their interaction with the simulator: (1) self-efficacy, (2) realism, and (3) educational value [14]. The questionnaire comprised 14 inquiries, with nine of them being structured according to a 5-point Likert scale and the remaining queries soliciting open-ended written responses. Prior to taking the survey, participants were required to provide informed consent by signing a form that outlined the study's procedures, potential risks, and benefits, providing participants with essential information. Participants were afforded ample time to decide whether to participate or not. Moreover, they were given the opportunity to seek clarification from the study team regarding any unclear aspects and to ensure their questions were addressed satisfactorily before agreeing to the terms outlined in the consent form.

Analyses

Quantitative Data

Descriptive statistics, including means and standard deviations, were used to analyze the numerical data.

Qualitative Data

Responses to open-ended questions were independently reviewed by two researchers to identify recurring themes. Each response was analyzed using a six-step procedure [15]. The process involved familiarizing with the responses, coding them, generating themes, reviewing themes, defining and naming the themes, and conducting the data analysis [15]. Themes were revised and refined, and any discrepancies were examined to ensure the validity of reliability of the analysis. 

## Results

The goal of the workshops, which used the two simulators co-developed in our laboratory, was to provide participants with hands-on experience in performing Z-plasty sutures and melanoma excisions and to determine whether these simulators were effective models for hands-on training of healthcare providers in R&R areas. 

Quantitative Data 

**Figure 5:**
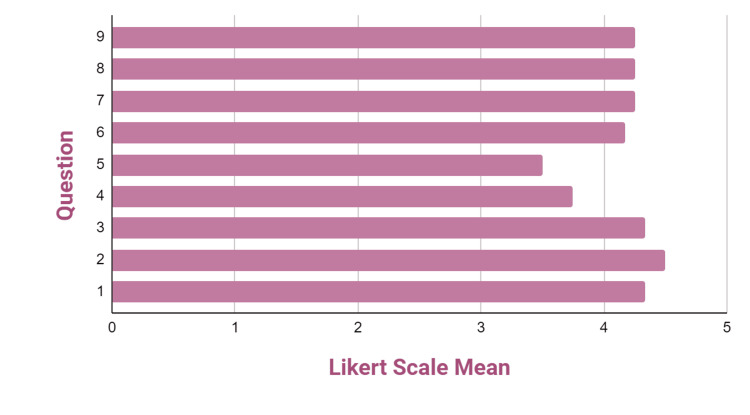
Likert scale means for questions regarding the Z-plasty simulator.

Z-plasty simulator: This simulator received an overall score of 4.03 on the 5-point Likert scale. Participants were asked to provide feedback on their interactions with the simulator, which was categorized into three main sections: (1) self-efficacy, (2) realism, and (3) educational value. Table 1 presents the frequencies, means, and standard deviations (SDs) of the quantitative responses provided by the workshop attendees, while Figure 5 illustrates these results graphically. In the self-efficacy section (questions 1-3), the simulator received a notably high average of 4.20, suggesting that participants felt confident in their ability to use the simulator. However, in the realism section (questions 4-7), the simulator received a lower average of 3.89, indicating that participants perceived it as less authentic. On the other hand, in the educational value section (questions 8 and 9), the simulator obtained a high score of 4.17, suggesting that participants viewed it as an effective training model for acquiring Z-plasty skills. Overall, the majority of the participants who completed the surveys expressed that the simulator is a viable option for training purposes. 

**Table 1:** Frequencies, means, and standard deviations of quantitative survey questions completed by workshop participants regarding the Z-plasty simulator.

Question	Likert scale frequencies						Mean	SD
	0	1	2	3	4	5		
1	-	-	-	-	10	3	4.22	0.44
2	-	-	-	2	8	3	4.08	0.63
3	-	-	-	1	7	5	4.31	0.62
4	-	-	3	2	7	2	3.56	1.02
5	-	-	3	5	4	2	3.36	1.01
6	-	1	-	1	8	4	4.00	1.04
7	-	-	-	-	8	6	4.43	0.51
8	-	1	-	-	8	5	4.13	1.03
9	-	-	1	-	8	5	4.21	0.80

**Figure 6:**
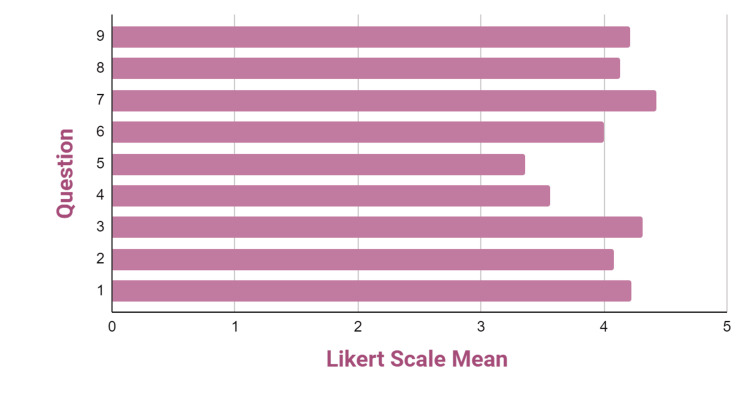
Likert scale means for questions regarding the melanoma simulator.

Melanoma simulator: This simulator received an overall score of 4.15 on the 5-point Likert scale. Participants provided feedback on their interaction with the simulator, which was categorized into three sections: (1) self-efficacy, (2) realism, and (3) educational value. Table 2 presents the frequencies, means, and SDs of the quantitative responses from the workshop participants, while Figure 6 illustrates these results graphically. In the self-efficacy section (questions 1-3), the simulator achieved a notably high average of 4.39 and an SD of 0.69, indicating confidence among participants in their ability to effectively engage with the simulator. In contrast, in the realism section (questions 4-7), the simulator obtained a lower average of 3.92 and an SD of 1.01, suggesting that participants perceived the simulator as less authentic. However, in the educational value section (questions 8 and 9), the simulator received a high score of 4.25 and an SD of 0.65, indicating that participants regarded it as an effective instructional model for acquiring melanoma excision skills. Overall, the majority of the participants who completed the surveys felt that the simulator could be utilized for training, although they also noted that some improvements would be beneficial. 

**Table 2:** Frequencies, means, and standard deviations of quantitative survey questions completed by workshop participants regarding the melanoma simulator.

Question	Likert scale frequencies						Mean	SD
	0	1	2	3	4	5		
1	-	-	-	1	6	5	4.33	0.64
2	-	-	-	1	4	7	4.50	0.66
3	-	-	-	2	4	6	4.33	0.78
4	-	-	3	-	6	3	3.75	1.14
5	-	-	3	3	3	3	3.50	1.17
6	-	-	-	2	6	4	4.17	0.72
7	-	-	-	2	5	5	4.25	0.74
8	-	-	-	1	7	4	4.25	0.61
9	-	-	-	1	7	4	4.25	0.61

Qualitative Data 

**Figure 7:**
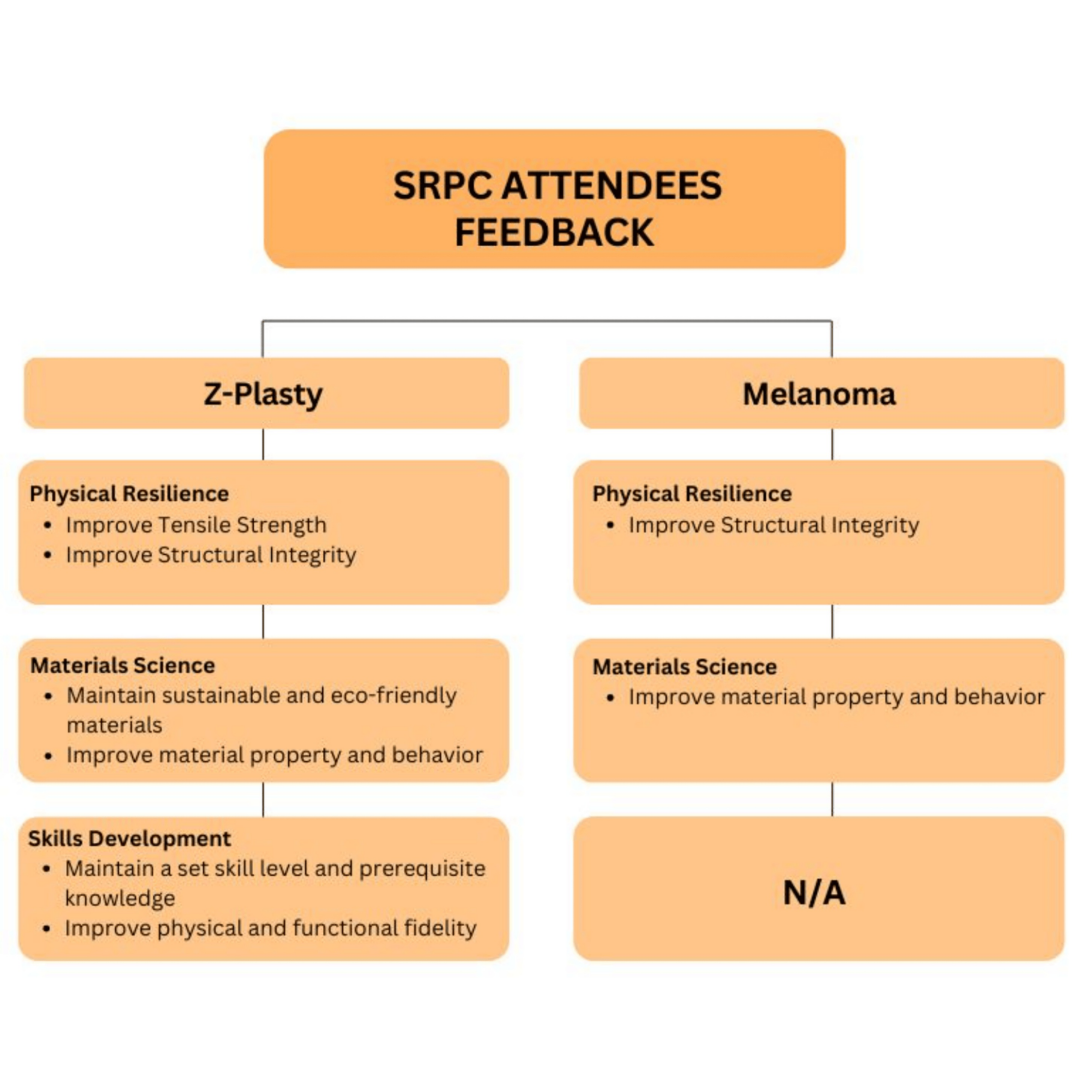
Feedback from the attendees of the Society of Rural Physicians of Canada (SRPC) workshop on Z-plasty and melanoma simulators.

For Z-plasty, three themes were identified: (1) physical resilience, (2) materials science, (3) and skills development (Figure 7). For melanoma, two main themes were identified: (1) physical resilience and (2) material science (Figure 7). 

Table 3 shows the supporting quotes for these themes. The first theme, physical resilience, refers to the simulator's ability to withstand the rigors of repeated and prolonged use while maintaining its structural integrity and functionality without any physical deterioration or malfunction. This attribute is essential to ensure that the simulator remains a reliable and effective tool for surgical education and practice. Participants using the Z-plasty simulator noted that it would be beneficial to increase the tensile strength of the model, as it would provide greater resistance to damage. They also emphasized the importance of improving the model's structural integrity, as it would be helpful if the model could maintain its shape without deformation. Participants using the melanoma simulator suggested reducing the toughness of the fibrous material to make the model less stiff and more flexible, which would enhance its realism. 

**Table 3:** Supporting quotes for themes of qualitative data.

Themes	Z-plasty supporting quotes	Melanoma supporting quotes
Physical resilience	“Increase tensile strength of plastic” (Participant 4). "Thread ripped threw simulator too easily” (Participant 12).	“Fibrous material is too tough” (Participant 4).
Materials science	“Did not use animals like pig’s feet” (Participant 13). "Stickiness of the base helps stabilize the simulator for suturing” (Participant 12). “Too tight and flat” (Participant 7). “Still quite rubbery. Does not mimic the layers of skin well” (Participant 8).	“Different textures for different skin layers” (Participant 12). "Love that you had different skin colors” (Participant 11).
Skills development	“Clarify skill level/ideal background knowledge needed to do advance level” (Participant 3). “Human tissue is not flat, and has curved surfaces. Too adherent, layers do not slide” (Participant 14).	—

The second theme, materials science, examines the simulator’s material properties and their impact on its functionality. Participants appreciated the Z-plasty model for its use of sustainable and eco-friendly materials to produce training simulators. They indicated that, with some refinements, the Z-plasty model could replace the unsustainable practice of using animal skins. Participants also recommended modifying the composition of the materials used in the Z-plasty pad. While the thickness of the base helped stabilize the model, they noted that reducing the rubbery texture of the model could help it better replicate the human skin. Participants using the melanoma simulator suggested incorporating different textures to represent the various layers of the skin. On the other hand, the use of diverse skin tones in the model was highly appreciated by the participants. 

The third theme, skills development, focuses on bridging knowledge gaps in surgical training. Participants indicated that skills development could be further enhanced by clarifying the required skill level and ideal background knowledge needed to effectively use the Z-plasty model. All participants emphasized the importance of improving the model's physical and functional fidelity, as they would like to see that the model closely mimics the human skin. They proposed several enhancements to improve the realism, such as incorporating skin layers, curved surfaces, weight, and pliability. These enhancements were seen as pivotal in advancing skills development, as a more authentic simulation would better replicate real-life scenarios, facilitating a more effective learning experience. 

## Discussion

The goal of the two workshops was to familiarize participants with the surgical procedures and instruments used in R&R practice. To accomplish this, the organizers and instructors of the workshops collaborated with our research and innovation laboratory to co-develop two simulators: the Z-plasty and melanoma simulators, which were used during the workshops for medical professionals. The simulators were designed and produced by maxSIMhealth (maxSIMhealth.com, Ontario Tech University, Oshawa, Ontario, Canada). Quantitative and qualitative data were collected post-workshop to evaluate the simulators and identify areas for future improvement.

AM has proven to be highly effective for use in SBE, as it allows for the customization of simulators to fit various contexts while also reducing costs [1]. For example, the Z-plasty simulator was produced for approximately $10 CAD, compared to $25 CAD for a similar alternative [16]. Likewise, the melanoma simulator was produced for around $12 CAD, whereas a comparable model costs over $300 CAD [17]. This approach prioritizes cost reduction by compromising some level of model realism, making simulators more accessible to learners [1]. The affordability of these simulators enables medical students and residents to purchase them for home use, providing additional opportunities for practice that can further refine their skills. Despite participants expressing a desire for increased realism, both simulators were still considered valuable tools for skills development.

The overall results were positive for both simulators. Following the utilization of the Z-plasty simulator, the majority of the responses to the System Usability Scale questionnaire expressed satisfaction. Participants found the simulator helped improve their ability to independently perform Z-plasty sutures, with many successfully suturing using the model. Other notable favorable responses included enhanced competence in performing Z-plasty suturing and recognition of the simulator as a valuable training tool for gaining knowledge in Z-plasty procedures. However, participants suggested increasing the tensile strength to prevent the thread from tearing through. They also noted that the simulator felt somewhat tight, flat, and rubbery and suggested improvements to better replicate the layers of human skin. 

A study investigating whether surgical residency could improve proficiency in a specific surgical procedure through brief practice sessions with feedback evaluated 37 junior surgical residents, who were assessed by attending plastic surgeons [18]. Following a five-minute practice session with feedback, the residents performed two flap Z-plasties on pig thighs using valid and reliable checklists and global grading scales [18]. The authors of this study found that training on a simple and understandable model, along with brief individualized practice and feedback, effectively improved residents' performance [18]. According to the findings, a five-minute practice session with a surgical trainee prior to performing a surgical procedure on a real patient could significantly improve surgical performance and outcomes [18]. This study shares similarities with the SRPC workshop, as both involved mentors assessing attendees' performance and providing constructive feedback to enhance their skills in performing Z-plasties [18]. However, a key distinction is that the study utilized a pig thigh model, which likely offered a more realistic training environment than the silicone-based simulator used in the SRPC workshop. Despite this, silicone-based simulators are more environmentally friendly, suggesting they could be a more sustainable long-term option. 

Participants also noted several beneficial outcomes using the melanoma simulator. They found it boosted their confidence in performing melanoma excisions and served as a valuable training model for gaining knowledge in this area. Additionally, participants successfully sutured using the simulator, which helped enhanced their skills, and they noted an improvement in their overall competence in performing melanoma excisions. However, the primary concern raised by the participants was the simulator's materials. The majority suggested that the textures should be adjusted to more closely resemble human tissues, as they found the current fibrous material to be too tough.

In comparison to another study, a major challenge in plastic surgery is altering the geometry and topology of the skin [19]. The surgeon’s specific decisions regarding the size and shape of the tissue to be removed and the subsequent closure of the wound can significantly impact the patient's quality of life after the procedure [19]. For patients diagnosed with malignant melanoma, the plastic surgeon often needs to resect the tumor and the surrounding area [19]. The study presented a comprehensive real-time virtual surgical environment built on finite element modeling, which simulated tissue cutting and manipulation [19]. The tool allowed the surgeons to make incisions, move tissue flaps, and create virtual sutures to simulate the closing of a skin defect [19]. The results indicated that the tool was effective, providing accurate physical simulations in an interactive environment that enhanced various aspects of their cognitive surgical practice [19]. However, our workshops provided hands-on practice with real simulators, which can often be more effective than virtual training. As stated in an article, the biggest challenge with virtual learning is the lack of realistic models that fully mimic tissues and respond accurately to surgical techniques [20].

A limitation identified during the workshop was the data collection methodology, which was not optimal due to time constraints. As a result, many participants were unwilling to complete surveys. This has a notable effect on the outcomes and conclusions as the survey responses were less detailed and may have been rushed due to the limited time. Consequently, this may have influenced the results and limited the depth of the feedback that was gathered. To address this issue in the future, adopting a "think out loud" protocol for data collection could be helpful [21]. This approach entails participants verbalizing their thoughts while performing a task, followed by an analysis of these spoken reflections. In contrast to other methods of collecting data, there are no interruptions or leading cues involved. Subjects are prompted to provide a real-time narrative of their thoughts, refraining from interpreting or explaining their actions and instead focusing solely on the task at hand. Another limitation was the small sample size. A long-term follow-up with participants who trained using the simulators could help assess whether their ability to handle melanoma and Z-plasty cases improved over time, leading to greater confidence and skill. 

Based on the findings, there are opportunities to improve the simulators for future use. Qualitative data highlighted the importance of enhancing the realism of the simulators. Participants suggested several improvements, including increasing the strength of materials, incorporating different textures to represent various skin layers, and adding features to better mimic real-life human characteristics. These improvements are crucial for creating realistic scenarios that participants may encounter in actual clinical situations. Simulation manikins that replicate lifelike details, such as veins, imperfections, hair, and aging across different ethnicities, would further enrich the training experience in healthcare, making it more authentic. This heightened realism in healthcare training allows learners to engage in a more realistic interaction similar to that with a real patient. However, an alternative view has been presented by Hamstra and Dubrowski [22], who argue that such features, while enhancing realism, may not be essential for skills acquisition.

## Conclusions

The fundamental melanoma and Z-plasty workshop offers vital training for healthcare professionals in R&R settings, helping them acquire and refine essential technical skills. Using 3D-printed melanoma and Z-plasty models for hands-on practice provides a practical and sustainable approach to skills development. These models provide significant advantages for current and future healthcare professionals, creating a safe and effective environment to refine crucial procedural skills. Additionally, the simulators serve as a means to enrich existing knowledge and foster a deeper comprehension of topics such as melanoma and Z-plasties. Feedback from participants, instructors, and observations during the workshop will inform enhancements for future SRPC conferences. By enhancing these simulators, the goal is to offer healthcare professionals and trainees an efficient and cost-effective means to practice Z-plasty and melanoma excision skills, ultimately improving clinical outcomes.

## Appendices

Supplemental Material 1 

**Table 4:** Quantitative and qualitative questions from the survey regarding the simulators rated by workshop participants . The quantitative questions were based on a 5-point Likert scale (1, strongly disagree, to 5, strongly agree). The qualitative questions were free-text.

Quantitative questions						
	Don’t know (.)	Strongly disagree (1)	Somewhat disagree (2)	Neutral (3)	Somewhat agree (4)	Strongly agree (5)
Self-efficacy						
The simulator helped improve my competence in performing Z-plasty suturing/melanoma excisions						
The simulator helped improve my confidence in performing z-plasty suturing/melanoma excisions						
The simulator helped improve my ability to perform z-plasty sutures/melanoma excisions independently						
Comments/suggestions regarding the simulator that may improve your self-efficacy						
Realism						
The simulator used has adequately realistic characteristics/features						
The simulator felt accurate						
The simulator was durable						
I was able to successfully suture using this simulator						
Comments/suggestions regarding the simulator to improve the fidelity during the training session						
Educational value						
The simulator is a good training tool for knowledge in Z-plasty suturing/melanoma excisions						
The simulator is a good training tool for skills in performing Z-plasty suturing/melanoma excisions.						
Comments regarding educational value						
Global: Please check one statement below with which you most agree. For evaluation of simulator: □ This simula(tor/tion) requires extensive improvements before it can be considered for use in training. □ This simula(tor/tion) requires minor adjustments before it can be considered for use in training. □ This simula(tor/tion) can be used in training, but should be improved slightly. □ This simula(tor/tion) can be used in training with no improvements made.						
Qualitative questions						
Comments regarding the simulator that may improve your self-efficacy						
Comments/suggestions regarding the simulator to improve the realism of the simulator						
Please suggest any changes you would make to the simulator						
What specific changes would you suggest to improve your learning experience? Please use the space provided below to describe if needed						
What alternative model have you used to train this skill (or what alternative model was used in this session), and how does it compare to this simulator? If no alternative was used, please write N/A						
